# Olfactory and cognitive decrements in 1991 Gulf War veterans with gulf war illness/chronic multisymptom illness

**DOI:** 10.1186/s12940-024-01058-2

**Published:** 2024-01-30

**Authors:** Linda L. Chao

**Affiliations:** 1 Departments of Radiology & Biomedical Imaging and Psychiatry & Behavioral Science, University of Calfiornia, 505 Parnassus Avenue, San Francisco, CA 94143 USA; 2 San Francisco Veterans Affairs Health Care System, 4150 Clement Street, San Francisco, CA 94121 USA

**Keywords:** Gulf war, Veterans, Cognition, Cognitive, Olfaction, neurotoxicant

## Abstract

**Background:**

Gulf War illness (GWI)/Chronic Multisymptom Illness (CMI) is a disorder related to military service in the 1991 Gulf War (GW). Prominent symptoms of GWI/CMI include fatigue, pain, and cognitive dysfunction. Although anosmia is not a typical GWI/CMI symptom, anecdotally some GW veterans have reported losing their sense smell shortly after the war. Because olfactory deficit is a prodromal symptom of neurodegenerative diseases like Parkinson’s and Alzheimer’s disease, and because we previously reported suggestive evidence that deployed GW veterans may be at increased risk for Mild Cognitive Impairment (MCI) and dementia, the current study examined the relationship between olfactory and cognitive function in deployed GW veterans.

**Methods:**

Eighty deployed GW veterans (mean age: 59.9  ±7.0; 4 female) were tested remotely with the University of Pennsylvania Smell Identification Test (UPSIT) and the Montreal Cognitive Assessment (MoCA). Veterans also completed self-report questionnaires about their health and deployment-related exposures and experiences. UPSIT and MoCA data from healthy control (HC) participants from the Parkinson’s Progression Markers Initiative (PPMI) study were downloaded for comparison.

**Results:**

GW veterans had a mean UPSIT score of 27.8  ± 6.3 (range 9–37) and a mean MoCA score of 25.3  ± 2.8 (range 19–30). According to age- and sex-specific normative data, 31% of GW veterans (vs. 8% PPMI HCs) had UPSIT scores below the 10th percentile. Nearly half (45%) of GW veterans (vs. 8% PPMI HCs) had MoCA scores below the cut-off for identifying MCI. Among GW veterans, but not PPMI HCs, there was a positive correlation between UPSIT and MoCA scores (Spearman’s ρ = 0.39, *p* < 0.001). There were no significant differences in UPSIT or MoCA scores between GW veterans with and without history of COVID or between those with and without Kansas GWI exclusionary conditions.

**Conclusions:**

We found evidence of olfactory and cognitive deficits and a significant correlation between UPSIT and MoCA scores in a cohort of 80 deployed GW veterans, 99% of whom had CMI. Because impaired olfactory function has been associated with increased risk for MCI and dementia, it may be prudent to screen aging, deployed GW veterans with smell identification tests so that hypo- and anosmic veterans can be followed longitudinally and offered targeted neuroprotective therapies as they become available.

## Background

Gulf War Illness (GWI), also known as Chronic Multisymptom Illness (CMI), is a multi-faceted condition estimated to affect 250,000 veterans of the 1990-91 Gulf War (GW) [1, 2]. More than thirty years after the end of the GW, many veterans still suffer GWI/CMI symptoms, which include a combination of persistent fatigue, musculoskeletal pain, sleep, gastrointestinal, and respiratory problems, skin rashes, and cognitive dysfunction. Although anosmia is not a typical GWI symptom, anecdotally some GW veterans have reported losing their sense smell shortly after the GW. Despite this, twenty years ago Vasterling and colleagues found no significant differences in olfactory and cognitive function when they compared deployed GW veterans with non-deployed GW era veterans [3].

In the years since the Vasterling et al. study was published, individual reports and meta-analyses of neuropsychological outcomes have documented significant cognitive impairments in veterans with GWI compared to healthy GW veterans [4, 5]. We previously found a higher-than-expected rate of Mild Cognitive Impairment (MCI), a clinical syndrome where cognitive impairment is greater than expected for one’s age, but not severe enough to meet diagnostic criteria for dementia [6], in a convenience sample of 200 + middle-aged (median age 52 years) GW veterans [7]. We recently replicated this finding in a larger cohort of 952 GW veterans [8]. Because MCI is more common among older (≥ 70 years) than middle-aged adults [9], this finding is consistent with the suggestion by Zundel and colleagues that GW veterans may be aging at a faster rate than the general population [10].

It has been well documented that olfactory function declines with age [11]. Research also suggests that poor olfaction is one of the earliest prodromal symptoms of neurodegenerative diseases such as Parkinson’s and Alzheimer’s disease [12–14]. Because impaired olfactory function has been associated with plaques and tangles in the olfactory bulb, entorhinal cortex, and hippocampus in autopsy studies [15], impaired olfaction may be a harbinger of MCI due to Alzheimer’s disease [16–19] or of other forms of dementia (e.g., Lewy body [20] and vascular dementia [21]).

The current study re-examined the relationship between olfactory and cognitive function in deployed GW veterans. Based on our previous reports that GW veterans with high levels of deployment-related exposures may be at increased risk for Parkinson’s disease (PD) [22], deployed GW veterans may be at increased risk for MCI [7, 8], and Zundel and colleagues’ suggestion that deployed GW veterans may aging more rapidly than their civilian counterparts [10], we hypothesized that deployed GW veterans would exhibit evidence of both olfactory and cognitive impairment.

## Methods

### Study participants

Participants were 80 deployed GW veterans recruited from 2020 to 2023 through the San Francisco VA Health Care System (SFVAHCS) as part of studies funded by the Department of Veterans Affairs (CX000798-05) and Department of Defense/Congressionally Directed Medical Research Programs (W81XWH-21-1-0656).

### Study design

The studies’ remote protocols were approved by the Institutional Review Boards of the University of California, San Francisco (UCSF), the San Francisco VA Health Care System (SFVAHCS), and the Department of Defense Office of Human and Animal Research Oversight. All participants provided informed consent electronically via VA DocuSign, completed self-report questionnaires about their health, GWI symptoms, and GW-related experiences remotely via REDCap, and participated in remote assessments of olfaction and cognition over Zoom.

### Measures

#### Self-report questionnaires

All participants completed the Kansa Gulf War Military History and Health Questionnaire [23] remotely via REDCap. We used the participants’ responses to this questionnaire to assess Kansas Gulf War Illness (GWI) case status [23], Centers for Disease Control and Prevention (CDC) Chronic Multisymptom Illness (CMI) case status [24], and deployment-related exposures. Additionally, participants completed a self-report questionnaire about whether they received COVID vaccination and/or had ever tested positive for COVID.

#### Olfactory function

The Pennsylvania Smell Identification Test (UPSIT) [25, 26], a standardized, forced-choice assessment of 40 odorants, was used to assess olfactory function remotely. As part of the UPSIT procedures, participants are asked if they suffer from smell and/or taste problems. For each odorant, participants were asked to select among 4 choices to identify the odorant presented. Scoring was based on the number of odorants correctly identified. We mailed UPSIT kits to participants and completed assessments with them over Zoom. Previous studies have demonstrated the feasibility of administering the UPSIT remotely [27, 28], and results from at-home UPSIT collection have been shown to be comparable to UPSIT data collected in clinic [29].

#### Cognition

The Montreal Cognitive Assessment (MoCA) [30] was used to test cognitive function remotely. The MoCA evaluates eight cognitive domains including short-term memory, visuospatial abilities, executive function, attention, concentration, working memory, language, and orientation to space and time. Remote administration of the MoCA has shown good validity relative to face-to-face administration [31] and has been validated in different populations [32, 33].

### Case status definitions

The Centers for Disease Control and Prevention (CDC) Chronic Multisymptom Illness (CMI) case status was operationalized as the presence of persistent symptoms over six months in two out of three domains: fatigue (lack of energy/overly tired), musculoskeletal pain (joint and/or muscle pain), and cognitive/mood disorders (e.g., difficulty remembering, difficulty concentrating, trouble sleeping, moodiness, and anxiousness) [24].

The Kansas Gulf War Illness (GWI) case status was operationalized as the presence of moderately severe or multiple mild chronic symptoms in at least three of six categories: fatigue/sleep problems, pain, neurological, cognitive and mood symptoms, respiratory complaints, gastrointestinal problems or skin symptoms [23]. Veterans were excluded as Kansas GWI cases if they reported any of the following conditions/diagnoses: Alzheimer’s disease (AD), Parkinson’s disease (PD), amyotrophic lateral sclerosis (ALS), multiple sclerosis (MS), lupus, seizure disorder, uncontrolled diabetes, heart disease other than hypertension, stroke, cancer in the previous three years, liver disease, kidney disease, chronic infections disease (e.g., hepatitis C), schizophrenia, bipolar disorder, or hospitalization for alcohol/drug dependence, depression, or posttraumatic stress disorder (PTSD) in the past two years [23]. Veterans were asked about these exclusionary conditions during the initial screening for the studies. Because having one or more Kansas GWI exclusionary conditions can be indicative of poor health, we show UPSIT and MoCA scores separately in veterans with Kansas GWI exclusionary conditions in the results section.

### Parkinson’s progression markers initiative (PPMI) healthy controls

We downloaded UPSIT and MoCA data from the Parkinson’s Progression Markers Initiative (PPMI) database (www.ppmi-info.org/data) in the preparation of this manuscript. Details of PPMI have been previously described (www.ppmi-info.org) [34, 35]. Briefly, PPMI is a multicenter, prospective study aimed at finding and identifying biomarkers for PD progression. PPMI recruits patients with PD, participants at risk for PD based on clinical features, genetics or other biomarkers (i.e., prodromal PD), and participants with no neurological disorder or first degree relatives with PD (i.e., healthy controls, HC). We used UPSIT and MoCA data from PPMI HCs as a reference for the GW veteran sample. Because PPMI includes participants 30 years and older, we only downloaded data from PPMI HCs who were 50 years and older to match the GW veteran cohort. Although we tried to match the PPMI HC sample to the GW veteran sample for age by only including PPMI HCs who were 50 years or older, the PPMI HC sample (mean age 65 years) was still older than the GW veteran sample (mean age 60 years). We were also unable to match the PPMI and GW veteran samples on ethnicity (2% Hispanic in PPMI vs. 10% among GW veterans), race (95% White in PPMI vs. 83% among GW veterans), education (mean 16 years in PPMI vs. mean 15 years among GW vetearns), or sex (36% female in PPMI vs. 5% among GW veterans). All PPMI participants provided informed consent. Ethical approval had been granted by institutional review boards or ethics committees. Enrollment of the PPMI HCs whose data were downloaded for this report occurred between February 2011 and August 2022. MoCA data from 249 PPMI HCs and UPSIT data from 64 PPMI HCs were accessed and downloaded from PPMI on November 28, 2023. All of the PPMI HCs who had UPSIT data also had MoCA data.

## Statistical analyses

Demographic characteristics of GW veterans are described through means and standard deviations (SD) for continuous variables and numbers and percentages for categorical variables. Comparisons between GW veterans and PPMI HCs were performed with the Wilcoxon Signed-Ranks test for continuous variables and chi-square tests for categorical variables. Associations between UPSIT and MoCA scores and deployment-related exposures were examined with Spearman’s Rank Order Correlations. All analyses were conducted with the Statistical Package for the Social Sciences (SPSS) Version 29.

## Results

### GW veteran demographics and characteristics

Characteristics of the GW veteran sample are summarized in Table 1.

**Table 1:** Summary demographic, military, and clinical characteristics of GW veteran sample

N	80
Age	59.9 (7.0)
No. (%) Male	76 (95%)
Years of education	14.5 (2.0)
Race Black/African American White/Caucasian Asian/Pacific Islander Other/Multiracial Missing/unreported	6 (8%) 66 (83%) 1 (1%) 6 (8%) 1 (1%)
Hispanic ethnicity	8 (10%)
Military Characteristics	
Enlisted during GW	70 (88%)
Branch of Service Army Air Force Marine Navy	41 (51%) 7 (9%) 16 (20%) 16 (20%)
Component of Service Active Duty Reserves National Guard	65 (81%) 11 (14%) 4 (5%)
CDC CMI cases	79 (99%)
Kansas GWI cases	38 (48%)
Had Kansas GWI exclusionary condition(s)	41 (51%)
Had COVID-19	35 (44%)
Had COVID-19 vaccine	53 (66%)
Mean MoCA score	27.8 (6.3)
Mean UPSIT score	25.3 (2.8)

Values are mean (SD) or N (%)

The GW veteran sample was predominately male (95%), approximately 60 years old with some post-high school education. All but one veteran met CDC CMI case status while 48% of the sample met Kansas GWI case status. A little over half of the sample (51%) had conditions that were exclusionary for the Kansas GWI case definition. These included poorly managed diabetes (*n* = 6), heart disease other than hypertension (*n* = 17), history of stroke (*n* = 4), rheumatoid arthritis (*n* = 9), cancer in the previous 3 years (*n* = 7), liver disease (*n* = 8), kidney disease (*n* = 7), chronic infectious disease (*n* = 2), and hospitalization within the previous 2 years for PTSD (*n* = 2), depression (*n* = 3), or substance abuse (*n* = 1). Because the GW veterans who did not meet Kansas GWI case status due to insufficient symptoms also had Kansas GWI exclusionary conditions or were CDC CMI cases,  none of the GW veterans in the sample could be considered “healthy controls.”

Because 99% of the GW veteran sample had CDC CMI, and because cognitive impairment is a symptom of CMI, we used MoCA and UPSIT data from PPMI HCs as a reference for “unimpaired” MoCA and UPSIT scores. The PPMI HCs who were during the initial phase of PPMI recruitment did not have cognitive impairment (i.e., MoCA scores ≥ 26) or current, active neurological disorders. Demographics of the PPMI HC sample are summarized in Table 2.

**Table 2:** Demographics of PPMI HC sample

	with MoCA	with MoCA + UPSIT
N	249	64
Age	65.0 (8.2)	67.1 (8.7)
No. (%) Male	160 (64%)	23 (36%)
Years of education	16.2 (3.1)	16.9 (3.2)
Race		
Black/African American	6 (2%)	5 (8%)
White/Caucasian	237 (95%)	55 (86%)
Asian/Pacific Islander	3 (1%)	3 (5%)
Other/Multiracial	1 (0%)	0 (0%)
Missing/unreported	2 (1%)	1 (2%)
Hispanic ethnicity	5 (2%)	0 (0%)

Values are mean (SD) or N (%)

The mean UPSIT score of the GW veteran sample was 27.8  ± 6.3 (range 9–37). According to existing olfactory diagnosis guidelines [26], 10% of the GW veterans’ UPSIT scores fell in the anosmia (loss of smell) range, 75% fell within the hyposmia (decreased sense of smell) range, while only 15% were in the normosmia (normal sense of small) range. In contrast, only 3% of PPMI HCs had UPSIT scores in the anosmia range while 47% had UPSIT scores in the normosmia range (see Table 3). The proportion of PPMI HC and GW veterans with UPSIT scores in the anosmia, hyposmia, and normosmia categories were significantly different (χ^2^ = 18.24, *p* < 0.001).

**Table 3:** Olfactory diagnosis classifications

	**GW Veterans**	**PPMI HC**
**Anosmia** - total loss of olfaction (UPSIT ≤ 18)	10%	3%
**Hyposmia** - decreased sensitivity (UPSIT 19–33, males; 19–34, females)	75%	50%
**Normosmia** - normal olfaction (UPSIT ≥ 34, males; ≥35 females)	15%	47%

**Fig. 1:**
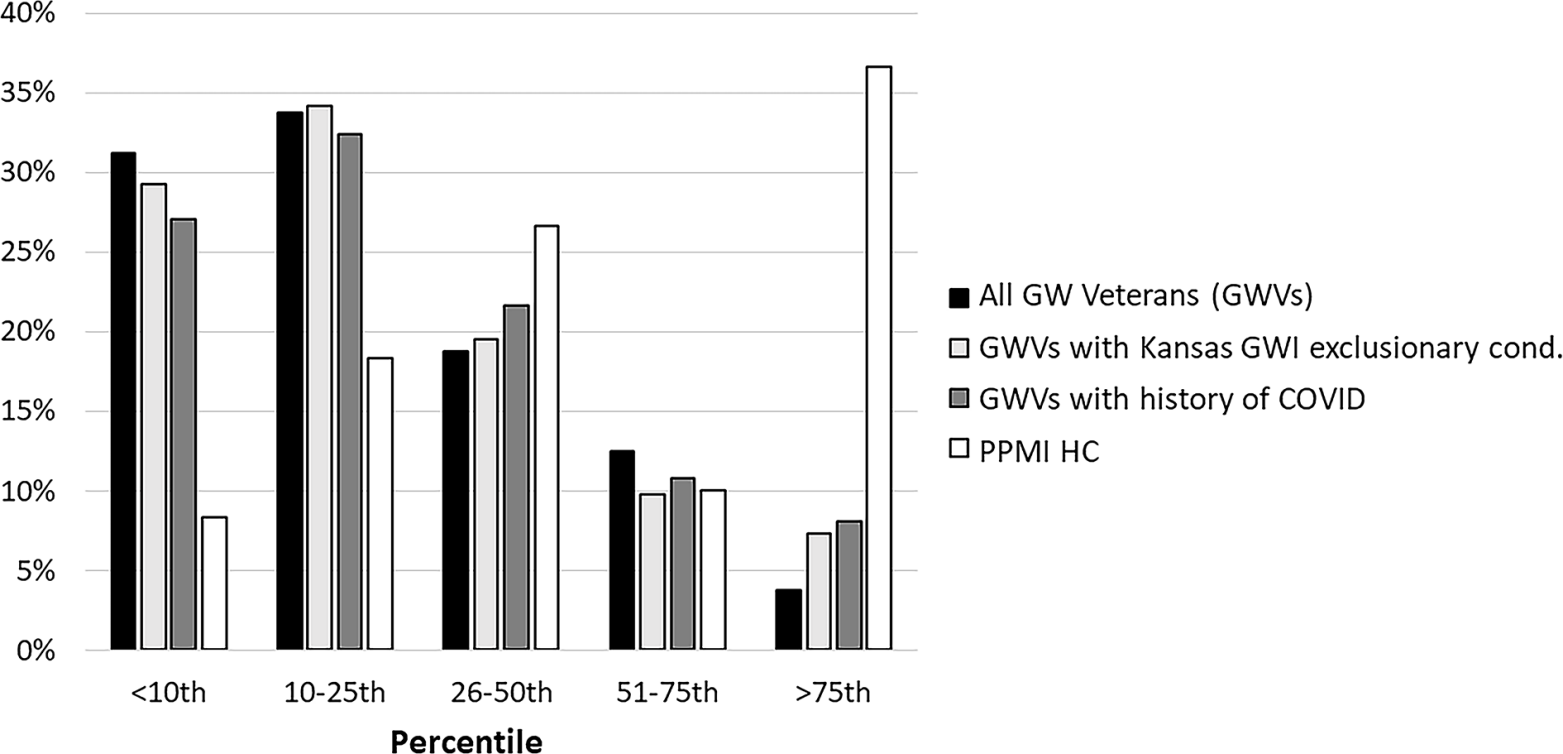
Percentage of GW veterans (black bars) and PPMI HCs (white bars) with UPSIT scores below the 10th percentile, in 10th to 25th, 26th to50th, 51st to 75th percentiles, and over the 75th percentile of sex- and age-specific normative UPSIT data. Subsets of GW veterans with Kansas GWI exclusionary condition(s) are represented in the light gray bars; subsets of GW veterans with history of COVID are represented in the dark gray bars

According to recently updated age-and sex-specific UPSIT normative data for adults 50 years and older [36], 31% of GW veterans had UPSIT scores below the 10th percentile while only 8% had UPSIT scores above the 75th percentile. Among PPMI HCs, only 8% had UPSIT scores below the 10th percentile of update age- and sex-specific UPSIT norms while 37% shad UPSIT scores above the 75th percentile (see Fig. 1). Kansas GWI exclusionary status (T = 1619.5, Z = 0.40, *p* = 0.69) and history of COVID (T = 1666, Z = 0.73, *p* = 0.47) did not significantly affect GW veterans’ UPSIT scores.

Consistent with prior research indicating that most people are inaccurate at accessing the nature and degree of their chemosensory problems [15, 37–41], the majority of the GW veterans in this study (69%) did not self-report problems with olfaction. Only 31% of the veterans self-reported an impaired sense of smell. The mean UPSIT scores of veterans with (25.6  ± 7.7) and without (28.8  ± 5.2) self-reported olfactory dysfunction was not significantly different (T = 847.5, Z = 1.72, *p* = 0.09).

**Fig. 2:**
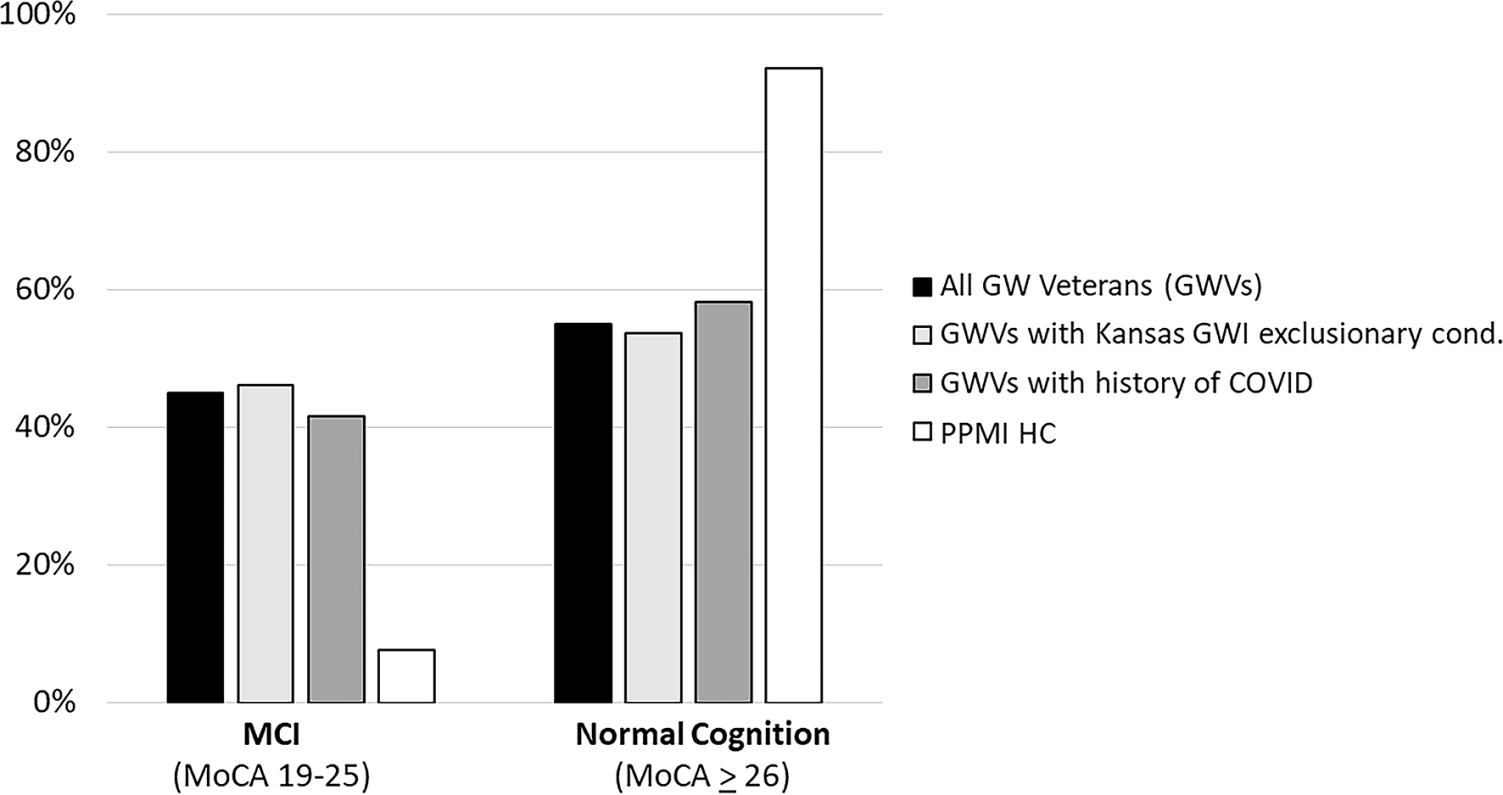
Percentage of GW veterans (black bars) and PPMI healthy controls (HCs, white bars) with MoCA scores in the MCI and Normal Cognition range. Subsets of GW veterans wit Kansas GWI exclusionary condition(s) are represented in the light gray bars; subsets of GW veterans with history of COVID are represented in the dark gray bars

The mean MoCA score in the GW veteran sample was 25.3  ± 2.8 (range 19–30). MoCA scores did not differ significantly as a function of Kansas GWI exclusionary status (T = 1482.5, Z = 0.58, *p* = 0.56) or history of COVID (T = 1590.5, Z = 0.69, *p* = 0.49). Nearly half (45%) of GW veterans had MoCA scores < 26, the recommended cut-off for identifying MCI [30]. In contrast, only 8% of PPMI HCs had MoCA scores < 26. The proportion of PPMI HC and GW veterans with MoCA scores in the MCI range was significantly different (χ^2^ = 59.81, *p* < 0.001, See Fig. 2).

**Fig. 3:**
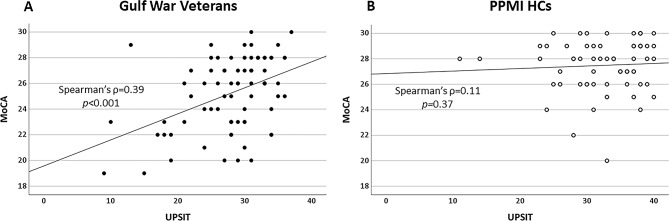
Scatter plot showing the correlation between UPSIT and MoCA scores in GW veterans (**A**) and PPMI HCs (**B**)

UPSIT and MoCA scores were positively correlated (Spearman’s ρ = 0.39, *p* < 0.001) in the GW veteran cohort. There was no significant relationship between UPSIT and MoCA scores in the PPMI HC cohort (Spearman’s ρ = 0.11, *p* = 0.37, see Fig. 3). There were no significant correlations between UPSIT scores, MoCA scores, or deployment-related exposures in GW veterans.

## Discussion

In this cross-sectional study, we found evidence of olfactory impairment in 80 deployed GW veterans, in accordance with our hypothesis. According to published olfactory diagnosis guidelines [26], 10% of the GW veterans had UPSIT scores indicative of anosmia while 75% had UPSIT scores indicative of hyposmia. According to recently updated sex- and age-specific normative data from 2 large cohorts of older (50+) community-dwelling volunteers, 31% of GW veterans scored below the 10th percentile on the UPSIT. In contrast, only 8% of PPMI HCs had UPSIT scores below the 10th percentile.

Consistent with prior research indicating that most people are inaccurate at accessing the nature and degree of their chemosensory problems [15, 37–41], the majority of GW veterans in this study (69%) did not self-reported problems with olfaction. Furthermore, UPSIT scores were comparable between the veterans who did and did not endorse olfactory problems. One study found evidence of greater cognitive impairment in individuals who were unaware of their olfactory dysfunction compared to those who were aware of their olfactory dysfunction and individuals with normal olfaction [41]. This may relate to the observation that MCI patients with greater cognitive decline tend to have less accurate self-awareness [42].

Twenty years earlier, Vasterling et al. investigated olfactory and cognitive function in deployed and non-deployed GW veterans and reported no significant differences between the two veteran groups [3]. Although the current study compared deployed GW veterans with CDC CMI to a US general population group (PPMI HCs), it is notable that the mean UPSIT score of deployed GW veterans in this study (28  ± 6) was 6–7 points lower than the mean UPSIT scores reported by Vasterling et al. for deployed (35  ± 3) and nondeployed (34  ± 3) GW veterans. This difference could be related to the fact that 99% of the GW veteran sample in this study had CDC CMI. Vasterline et al. did not report if any of the GW veterans in that study were CDC CMI cases. The difference could also be related to the age. Age-related decrements in olfaction have been well documented [11, 43]. Because the Vasterling et al. study was conducted 20 years earlier, the veterans in that study (~ 40 years old) were approximately 20 years younger than the GW veterans who participated in this study (~ 60 years old). Further evidence in support of this idea comes from is the finding that mean UPSIT scores of the PPMI HCs (32  ± 7), who were approximately the same age as the GW veterans in the current sample, was also lower than the UPSIT scores of deployed and non-deployed GW veterans in the Vasterline et al. study [3]. In light of the suggestion that deployed GW veterans may be aging more rapidly than the general population [10], it would be interesting to re-test the olfactory function of deployed GW veterans who participated in the 2003 Vasterling et al. study.

Another potential explanation for why the GW veterans in this study had poorer olfaction than the GW veterans in the Vasterling et al. study may be biological sex: There are well-documented sex differences in human olfaction [44], and the Vasterling et al. study had more female veterans (13% deployed GW veterans, 18% non-deployed GW veterans) than the current study (only 5%).

Health status may be a third reason why the GW veterans in this study had poorer olfaction than the GW veterans in the Vasterling et al. study. In addition to being 20 years younger, the deployed GW veterans in the Vasterling et al. study were likely healthier than the GW veterans in this study because that study had more restrictive exclusionary conditions, which included history of head trauma (i.e., loss of consciousness > 15 min or requiring medical care), central nervous system disease such as cerebrovascular disease, and medical disorders thought to affect smell, such as chronic respiratory illness. Although none of the veterans in this study had severe traumatic brain injury (TBI), some had mild-to-moderate TBI. There have been reports that head injury can affect olfactory function [45]. Furthermore, 51% of the veterans in the present study had conditions that are exclusionary for the Kansas GWI case status. None of the veterans had Alzheimer’s disease, Parkinson’s disease, amyotrophic lateral sclerosis, multiple sclerosis, lupus, or seizure disorder, but some of the veterans did have poorly managed diabetes, heart disease other than hypertension, liver disease, kidney disease, chronic infection, and history of stroke, cancer and had been hospitalized for alcohol/drug dependence, depression, and PTSD within two years of study participation. Although there were no significant differences in the UPSIT scores of GW veterans who did and did not have Kansas GWI exclusionary condition(s) in the current study, these conditions undoubtedly make the current veteran sample less healthy than the GW veterans who took part in the Vasterling et al. study.

Because the Vasterling et al. study took place 20 years earlier than this study, another major difference between this and the former study is the SARS-CoV-2 (COVID-19) pandemic. Olfactory dysfunction is a hallmark symptom of COVID-19 disease resulting from the SARS-CoV-2 virus [46]. Forty-four percent (44%) of the veterans in the current study reported having had or testing positive for COVID, although there were no significant differences in UPSIT scores of GW veterans who did and did not have a history of COVID.

Olfactory function has been proposed to be serve as a sensitive indicator of neurotoxic exposure [47, 48]. This is the reason why Vasterling et al. examined olfactory function in GW veterans in the earlier study, as a means of exploring the possible neurotoxic sequelae to GW deployment. However, contrary to their hypothesis, Vasterling et al. did not find differences in the UPSIT scores of deployed GW veterans with high versus low levels of GW-related exposures. We also failed to find a significant relationship between UPSIT scores and self-reported measures of GW-related exposures. Although the olfactory neuroepithelium is vulnerable to the toxic effects of environmental exposures [47, 49], olfactory sensory neurons have the power to regenerate [50]. Because it is likely that the olfactory sensory neurons regenerates throughout the lifetime [51], this may explain why Vasterling et al. and we were unable to detect a relationship between GW-related exposures and olfactory function in deployed GW veterans 10–30 years after the exposures occurred.

Olfactory function has also been proposed to be serve as a sensitive indicator of cognitive decline [18, 52–57]. This is because some of the same neuroanatomical structures, particularly in the hippocampal/limbic regions, subserve both cognitive (i.e., memory, attention, and processing speed) and olfactory function [58, 59]. In fact, there is growing evidence that olfactory *dys*function may be a harbinger of cognitive decline, including the transition for normal aging to MCI [12, 16, 60]. Therefore, it is compelling that there was a significant, inverse relationship between UPSIT and MoCA scores in GW veterans.

Nearly half (45%) of GW veterans had MoCA scores that met the cut-off for MCI [30]. This may related to the fact that all but one veteran in the current sample met CDC CMI case status. Cognitive dysfunction is a common symptom of CMI [4, 5, 61] and one of the defining symptom categories for CDC CMI. Reminiscent of our reports of higher-than-expected rates of MCI among middle-aged GW veterans [7, 8], this finding may suggest that deployed GW veterans, particularly those with CDC CMI, are at increased risk for future neurodegenerative diseases.

Neurodegeneration patterns in AD and Lewy body diseases, including PD, often begin in the olfactory bulb [62]. It has been suggested that damage to the olfactory blub from viral infection or an inflammatory response may be the catalyst for neurodegeneration in vulnerable individuals [63]. Olfactory dysfunction is a common sign of neuroinflammation of the central nervous system [64], which has been proposed to be the process that mediates olfactory loss, cognitive decline, and neurodegeneration [65, 66]. It is noteworthy that neuroinflammation has also been implicated in the pathophysiology of GWI [67, 68].

This study has some limitations that warrant consideration. First, because we did not have a GW veteran control group, we cannot say whether olfactory deficits are linked specifically to CMI/GWI case status or to GW deployment in general. Because the focus of this study was to not to characterize GWI per se, but rather to examine the cognitive and olfactory function of deployed GW veterans, particularly in relation to prognosis for MCI and dementia [7, 8], we felt justified in including as many GW veterans as possible. However, future studies will be necessary to determine if olfactory deficits are present in healthy GW veterans who do not have CMI/GWI. Second, this study included veterans with Kansas GWI exclusionary conditions. The rational for having exclusionary conditions for the Kansas GWI case definition was to avoid confounds from co-morbid conditions might produce symptoms similar to GWI or might interfere with respondents’ perception or reports of their symptoms [23]. However, 30 years after the war, GW veterans are aging, possibly at a faster rate than the general population [10], and developing age-related co-morbidities. Therefore, excluding GW veterans with Kansas GWI exclusionary conditions from research studies may result in a non-representative sample of GW veterans. Using VA electronic health records, we recently reported evidence that GW veterans with Kansas GWI exclusionary conditions had higher frailty indices than GW veterans without Kansas GWI exclusionary conditions [69]. Third, this study had a cross-sectional design. Future longitudinal studies will be necessary to determine the prognosis of the GW veterans who were classified as anosmic and hyposmic. Fourth, we did not have information about the GW veterans’ apolipoprotein (*APOE*) ɛ4 status. There have been reports that individuals with the *APOE* ɛ4 allele are at increased risk for MCI and AD [70, 71], and show deficits in olfactory function [72–75]. These limitations notwithstanding, results from this study suggest that deployed GW veterans with CDC CMI have impaired olfactory function. This is worrisome because impaired olfactory function has been associated with plaques and tangles in the olfactory bulb, entorhinal cortex, and hippocampus in autopsy studies [15]. Thus, impaired olfactory function may be indicative of MCI due to AD [16–19] or other forms of dementia (e.g., Lewy body [20] and vascular dementia [21]).

## Conclusion

This cross-sectional study documented evidence of impaired olfactory and cognitive function in a sample of deployed GW veterans with CDC CMI. Because impaired olfaction has been suggested to be a harbinger of future dementia, it may be prudent to screen GW veterans with non-invasive smell identification tests as they age, to clinically follow the veterans with hypo- and anosmia, and offer them neuroprotective therapies as they become available.

## Data Availability

No datasets were generated or analysed during the current study.

## Abbreviations

AD: Alzheimer’s Disease
ALS: Amyotrophic Lateral Sclerosis
CDC: Centers for Disease Control and Prevention
CMI: Chronic Multisymptom Illness
GW: Gulf War
GWI: Gulf War Illness
HC: Healthy Control
MCI: Mild Cognitive Impairment
MoCA: Montreal Cognitive Assessment
MS: Multiple Sclerosis
PD: Parkinson’s Disease
PPMI: Parkinson’s Progression Markers Initiative
SFVAHCS: San Francisco VA Health Care System
SPSS: Statistical Package for the Social Sciences
TBI: Traumatic Brain Injury
UCSF: University of California, San Francisco
UPSIT: University of Pennsylvania Smell Identification Test
VA: Veterans Affairs
